# Overexpression of CARM1 in breast cancer is correlated with poorly characterized clinicopathologic parameters and molecular subtypes

**DOI:** 10.1186/1746-1596-8-129

**Published:** 2013-08-02

**Authors:** Hongxia Cheng, Yejun Qin, Hui Fan, Peng Su, Xiaofang Zhang, Hui Zhang, Gengyin Zhou

**Affiliations:** 1 Department of Pathology, Shandong University School of Medicine, 44#, Wenhua Xi Road, Jinan, Shandong 250012, People’s Republic of China; 2 Department of Pathology, Provincial Hospital Affiliated to Shandong University, 324#, Jing 5 Rd, Jinan, Shandong 250021, People’s Republic of China

**Keywords:** CARM1, Breast cancer, Clinicopathologic parameters, HER2, Molecular subtype

## Abstract

**Background:**

Coactivator-associated arginine methyltransferase 1 (CARM1) belongs to the protein arginine methyltransferase family. CARM1 has been reported to be associated with high grade tumors in breast cancer. It still remains unknown the expression pattern of CARM1 in breast cancer and its relationships with clinicopathological characteristics and molecular subtypes.

**Methods:**

Two hundred forty-seven invasive breast cancer cases were collected and prepared for tissue array. There were thirty-seven tumors with benign glandular epithelium adjacent to the tumors among these cases. Molecular subtype and CARM1 expression were investigated using immunohistochemistry.

**Results:**

Cell staining was observed in the cytoplasm and/or nucleus. Staining for CARM1 was significantly stronger in adenocarcinoma compared with adjacent benign epithelium. There is a significant correlation between CARM1 overexpression with young age, high grade, estrogen receptor (ER) and progesterone receptor (PR) negative, increased p53 expression, and high Ki-67 index. Our study demonstrated CARM1 overexpression was associated with an increase in the protein expression of HER2. Furthermore, our data indicated CARM1-overexpression rate were remarkably higher in HER2 subtype (69.6%), luminal B subtype (59.6%) and TN subtype (57.1%) compared with luminal A subtype (41.3%).

**Conclusions:**

CARM1 expression was increased in invasive breast cancer. CARM1 overexpression was associated with poorly characterized clinicopathologic parameters and HER2 overexpression. There were significant differences between different molecular subtypes in their relationship to CARM1 overexpression. Our results support the value of using CARM1 in prognostic stratification of breast cancer patients and its potential therapeutic implications in targeting treatment.

**Virtual slides:**

The virtual slide(s) for this article can be found here: http://www.diagnosticpathology.diagnomx.eu/vs/4116338491022965

## Background

Breast cancer is the most common cancer of woman in many countries [1]. Human breast carcinomas represent a collection of diverse tumors that vary in their natural history and responsiveness to therapy. Because of its heterogeneity, many tumor tissue biomarkers have been identified and used in breast cancer diagnosis to classify subsets, indicate specific therapies and predict tumor behavior. Today, biomarkers such as estrogen receptor (ER), progesterone receptor (PR), p53, Ki-67 and human epidermal growth factor receptor type 2 (HER2) guide treatment decisions and prognosis.

HER2 is a proto-oncogene and is a member of the *HER* gene family, which includes HER1 (epidermal growth factor receptor, EGFR/erbB1), HER2, HER3 (erbB3) and HER4 (erbB4). The receptor is amplified or overexpressed (or both) in approximately 18–20% of breast cancers [2]. HER2 promotes cell proliferation and angiogenesis and inhibits apoptosis via several pathways [2], and HER2-positive status is a negative prognostic factor [3,4]. Treating HER2-positive breast cancer with anti-HER2 monoclonal antibodies, such as trastuzumab, has markedly improved the outcome of this disease [5]. One major challenge to targeted therapy, however, is acquired and primary resistance. Acquired resistance eventually develops in most patients in the advanced disease setting [6]. Advances in molecular biology have led to the identification of potential markers of prognostic and therapeutic importance in breast cancers.

Methylation of histones by protein arginine methyltransferases (PRMTs) is increasingly being acknowledged as an important aspect for the dynamic regulation of gene expression. CARM1 (coactivator-associated arginine methyltransferase 1) is a kind of type I protein arginine methyltransferase that catalyzes the formation of asymmetric dimethylarginine [7]. It initially was described as a transcriptional activator of the p160 family of nuclear receptor-associated proteins [8]. The p160 family includes steroid receptor coactivators-1(SRC-1), SRC-2 and SRC-3/AIB1 (amplified in breast cancer 1) [8]. CARM1 functions as a coactivator for many nuclear receptors (NRs) [9,10], including ERα [11]. ERα plays a pivotal role in promoting the proliferation of several types of estrogen-stimulated breast cancer [12]. CARM1 has also been shown to be a molecular switch that controls multiple classes of gene-specific transcription factors, including p53, NF-κB, LEF1/TCF4, E2Fs, and cyclin E1 [9,13-16]. These suggest this enzyme plays pleiotropic roles in cell proliferation and survival. Some researchers had investigated the expression of CARM1 in many kinds of malignant tumors [17-19]. Aberrant expression of CARM1 has been linked to human breast cancer tissue in a few reports [13,16,17]; however, current studies are contradictory and incomplete. The mRNA level of CARM1 was found to be elevated in grade 3 breast tumors in a cohort of 81 human breast carcinomas of various types [16]. While another study demonstrated there was inverse correlation between CARM1 expression and tumor grade in ER + and LN-breast cancer cases [13]. Kim YR et al. reported CARM1 overexpression was noted only in small number of breast cancer patients (27%) [17]. All these reports suggest CARM1 is an important factor involved in progression and may affect prognostication of breast cancer. However, many of these studies were limited either by low n values of breast cancer patients or by a special tumor type. It still remains unclear whether CARM1 expression is correlated with clinicopathological features, molecular subtype and prognosis.

The aim of this study was to characterize the CARM1 expression pattern in invasive breast carcinoma and to analyze its relationship with clinicopathologic characteristics, including the expression of ER, PR, HER2, p53 and Ki-67 index. Additionally, we compared the expression of CARM1 in different molecular subtypes to assess its potential value in improving patient stratification and guiding personal patient management.

## Methods

### Tumor samples and clinical material

Two hundred forty-seven untreated breast tumor samples were collected from the pathology department at Provincial Hospital and Qilu Hospital affiliated to Shandong University from March 2007 to March 2009. There were thirty-seven tumors with benign glandular epithelium adjacent to the tumors. Tumor tissue was collected and processed for immunohistochemistry using a tissue microarray. All the diagnoses were made by two pathologists following the WHO Classification of Tumors of the Breast (2012) [20]. Lymph node metastases were present in 119 patients (48.6%) at the time of surgery; these metastases were detected in the final paraffin sections. Tumor stage was based on American Joint Committee on Cancer (AJCC) TNM staging system [21], and histological grade used the Elston and Ellis modification of the Scarff-Bloom-Richardson grading system [22]. Estimations of tumor grade were scored simultaneously by two investigators blinded to the patient’s clinical findings. In case of different views, a third independent investigator provided an opinion. Patients with the following characteristics were excluded from this study: patients who accepted therapy before surgery; tumor histology other than invasive ductal or lobular carcinoma; cases with incomplete data on ER, PR, or HER2. For the use of these clinical materials for research purposes, prior patient content and approval from the Institutional Research Ethics Committee of our hospitals were obtained.

### Immunohistochemistry

All 247 breast samples were fixed in formalin and embedded in paraffin wax. Immunohistochemical staining was performed using the streptavidin peroxidase complex method on 4 μm thick sections. After being deparaffinized with xylenes and rehydrated, sections were submerged into 10 mM citrate buffer (pH 6.0) and microwaved for 15 min to retrieve the antigens, followed by incubation in 3% H2O2 for 10 minutes to quench endogenous peroxidase. Nonspecific binding of antibodies was inhibited by incubation in 5% normal goat serum. Rabbit anti-CARM1 antibody (dilution 1:50, Upstate Biotechnology, Lake Placid, NY) was incubated with the sections overnight at 4°C; the second antibody was from SP reagent kit (Zhongshan Biotechnology Company, Beijing, China). After washing, tissue sections were incubated with biotinylated anti-rabbit secondary antibody, and then incubated in streptavidin-peroxidase complex. In addition, the sections were stained with diaminobenzidine (DAB) and counterstained with hematoxylin. For negative controls, sections were incubated in PBS instead of the primary antibody. We also used a control IgG to prove that there were no non-specific reactions of the CARM1 antibody.

### Assessment of immunohistochemical staining

The immunostained slides were evaluated by two pathologists independently in a blind fashion. Immunohistochemistry scores of CARM1 derived from the assessment of both staining intensity and percentage of positive cancer cells as described previously [23]. Nuclear and plasmid staining was scored separately. Intensity of staining was grade as 0–3(0, no staining; 1, weak staining; 2, unequivocal moderate staining; and 3, strong staining). The percentage of positive staining cells was recorded in increments of 10%, from 0 to 100%. These two scores were multiplied to generate an immunohistochemical score (IHC score) generate with a range of 0–300. Positive staining for ER, PR and p53 was defined as staining of >10% of nuclei,and an elevated Ki-67 index was also defined as >10% tumor cells with distinctly positive nuclear staining. HER2-positivity was defined as 3(+) on IHC staining or HER2 gene amplification by fluorescence in situ hybridization (FISH). FISH was performed selectively when the score was 2(+).

### Classification of molecular subtypes

In breast cancer, utilization of immunohistochemistry as a surrogate for molecular classification by gene expression profiling has been used in a number of large population-based studies and has been shown to provide an acceptable level of accuracy for determining molecular phenotype [24,25]. Different subtypes of tumors were shown to have different tumor biology, different prognoses, and different responses to therapy. Molecular subtypes were approximated using histological grade and the ER, PR, and HER2 status of the primary tumor [26]. Cases that were ER-positive and/or PR-positive, HER2-negative and either histologic grade 1 or 2 were classified as luminal A cancers; cases that were ER-positive and/or PR-positive and HER2-positive, or ER-positive and/or PR-positive, HER2-negative and histologic grade 3 were classified as luminal B cancers; cases that were ER-negative, PR-negative, and HER2-positive were classified as HER2 type; and cases that were negative for ER, PR, and HER2 were classified as triple negative(TN).

### Analysis

Statistical analysis was carried out using SPSS 11.0. The statistical analysis of CARM1 expression between benign and tumor tissue was performed with the one-way ANOVA. The correlation between CARM1 expression and clinicopathological parameters, CARM1 expression and universal biological factors, and CARM1 expression in different subtypes were examined using chi-square test or Fisher’s exact test if appropriate. Bivariate correlations between study variables were calculated by Spearman’s rank correlation coefficient. Positive nuclear and cytoplasmic staining for CARM1 was estimated separately. Differences were considered statistically significant for *p* values < 0.05.

## Results

### Expression of CARM1 in breast invasive carcinoma

**Figure 1:**
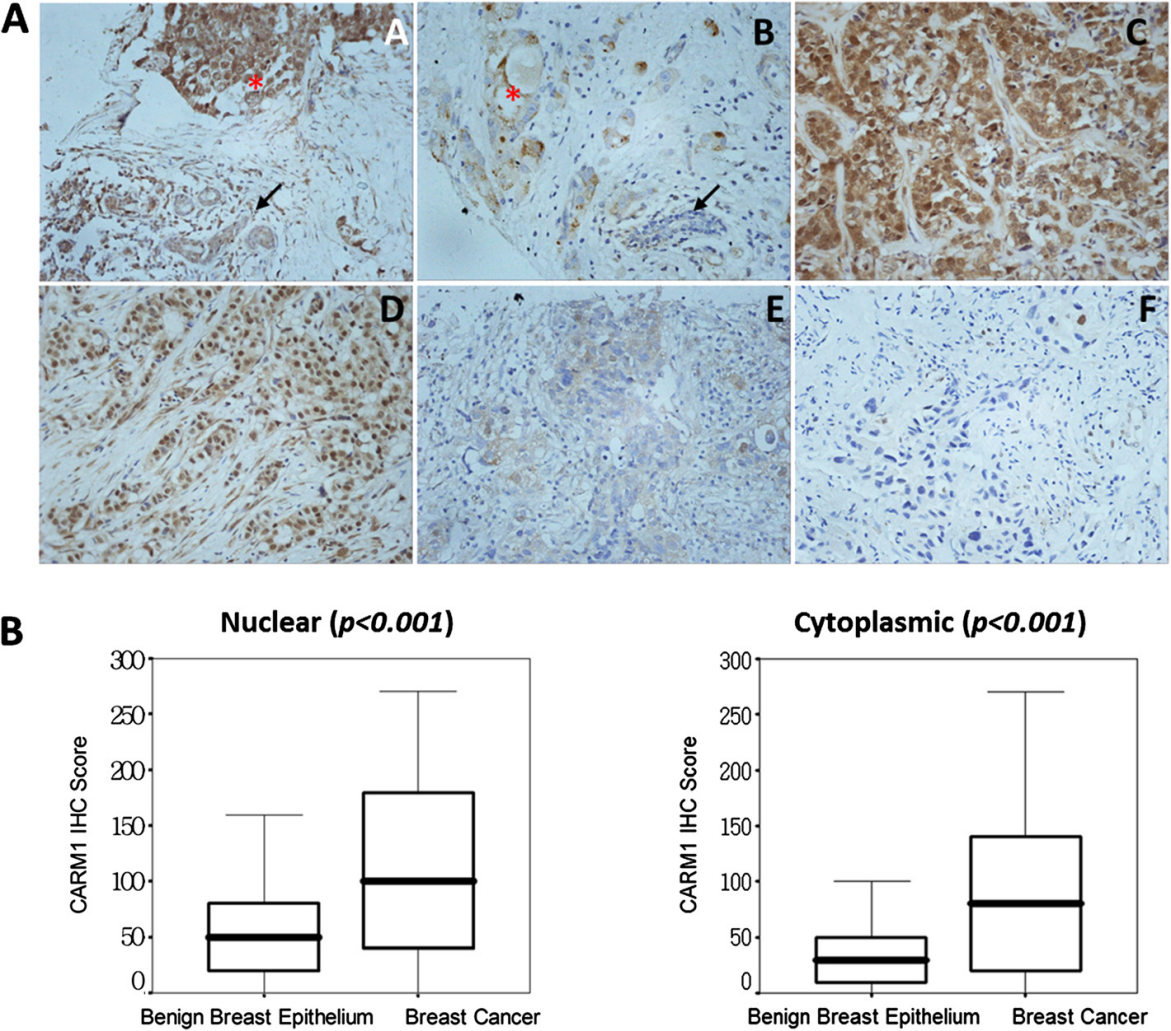
**The expression pattern of CARM1 in human invasive breast cancer (×400). A** In positive samples, the expression of CARM1 in adenocarcinomas was considerably increased compared with the adjacent benign glandular epithelium **(A and B)**. The increased expression was both nuclear and cytoplasmic **(C)**, predominantly nuclear **(D)**, or predominantly cytoplasmic **(E)**. A representative negative case is shown **(F)**. Asterisks indicate adenocarcinoma. Arrows indicate benign breast epithelium. **B** CARM1 expression was increased significantly in invasive breast cancer compared with benign epithelium (one-way ANOVA P < 0.001).

To determine the pattern of CARM1 expression in invasive breast cancer, tissue microarrays containing cancer tissue with several adjacent benign tissues were used for immunohistochemical staining of CARM1. In the 37 tumors with matching benign tissues adjacent to the tumors, most benign breast epithelial cells exhibited negative or weak staining for CARM1 (mean IHC score, nuclear 50.00, cytoplasm 37.57); staining for CARM1 was significantly stronger in matched adenocarcinoma cells (mean IHC score, nuclear 111.35, cytoplasm 87.57). The increased expression was observed in the cytoplasm and/or the nucleus (Figure 1). The differences between malignant tissue specimens and benign epithelium were statistically significant (one-way ANOVA, nuclear *p* < 0.001, cytoplasmic *p* < 0.001, Figure 1). CARM1 expression in breast invasive carcinoma showed nuclear and/or cytoplasmic staining patterns, and each was scored separately. In positive samples, a large number of cases were stained in the nucleus and cytoplasm, while some cases showed either mainly nuclear or cytoplasmic staining pattern (Figure 1). Our results indicated 126 of 247 (51.0%) patients showed nuclear positive and 142 of 247 (57.5%) showed cytoplasmic positive (Tables 1 and 2).

**Table 1:** Correlation between nuclear CARM1 expression and the clinicopathologic characteristics of breast cancer patients

**Clinicopathologic characteristics**	**n**	**Nuclear CARM1 expression**		***P*﹡**
		**Negative or low expression (IHC score < 80)**	**High or over expression (IHC score f ≥ 80)**	
Age(y)				
≤40	53	16 (30.2)	37 (69.8)	0.002
>40	194	105 (54.1)	89 (45.9)	
Tumor size				
≤2 cm	125	65 (52.0)	60 (48.0)	0.338
>2 cm	122	56 (45.9)	66 (54.1)	
Tumor number				
1	234	121 (51.7)	113 (48.3)	<0.001
≥2	13	0 (0)	13 (100.0)	
Grade				
I&II	181	96 (53.0)	85 (47.0)	0.035
III	66	25 (37.9)	41 (62.1)	
Lymph node metastasis				
N0	127	56 (44.1)	71 (55.9)	0.113
N1, N2,N3	120	65 (54.2)	55 (45.8)	
TNM stage				
I	74	36 (48.6)	38 (51.4)	0.886
II	111	53 (47.7)	58 (52.3)	
III	62	32 (51.6)	30 (48.4)	
ER				
negative	90	35 (38.9)	55 (61.1)	0.016
positive	157	86 (54.8)	71 (45.2)	
PR				
negative	136	56 (41.2)	80 (58.8)	0.007
positive	111	65 (58.6)	46 (41.4)	
HER2				
negative	206	107 (51.9)	99 (48.1)	0.037
positive	41	14 (34.1)	27 (65.9)	
Expression of Ki67				
low (≤10%)	55	35 (63.6)	20 (36.4)	0.014
high (>10%)	192	86 (44.8)	106 (55.2)	
Expression of P53				
low	95	57 (60.0)	38 (40.0)	0.006
high	152	64 (42.1)	88 (57.9)	

﹡ *P* value is the result of χ^2^ test in the same factor, and < 0.05 is considered statistically significant.

**Table 2:** Correlation between cytoplasmic CARM1 expression and the clinicopathologic characteristics of breast cancer patients

**Clinicopathologic characteristics**	**n**	**Cytoplasmic CARM1 expression**		***P*﹡**
		**Negative or low expression (IHC score < 80)**	**High or over expression (IHC score ≥ 80)**	
Age(y)				
≤40	53	16 (30.2)	37 (69.8)	0.041
>40	194	89 (45.9)	105 (54.1)	
Tumor size				
≤2 cm	125	57 (45.6)	68 (54.4)	0.320
>2 cm	122	48 (39.3)	74 (60.7)	
Tumor number				
1	234	101 (43.2)	133 (56.8)	0.379
≥2	13	4 (30.8)	9 (69.2)	
Grade				
I&II	181	85 (47.0)	96 (53.0)	0.019
III	66	20 (30.3)	46 (69.7)	
Lymph node metastasis				
N0	127	48 (37.8)	79 (62.2)	0.123
N1, N2,N3	120	57 (47.5)	63 (52.5)	
TNM stage				
I	74	30 (40.5)	44 (59.5)	0.384
II	111	44 (39.6)	67 (60.4)	
III	62	31 (50.0)	31 (50.0)	
ER				
negative	90	28 (31.1)	62 (68.9)	0.006
positive	157	77 (49.0)	80 (51.0)	
PR				
negative	136	50 (36.8)	86 (63.2)	0.043
positive	111	55 (49.5)	56 (50.5)	
HER2				
negative	206	93 (45.1)	113 (54.9)	0.060
positive	41	12 (29.3)	29 (70.7)	
Expression of K-i67				
low (≤10%)	55	31 (56.4)	24 (43.6)	0.018
high (>10%)	192	74 (38.5)	118 (61.5)	
Expression of P53				
low	95	45 (47.4)	50 (53.6)	0.222
high	152	60 (39.5)	92 (60.5)	

﹡ *P* value is the result of χ^2^ test in the same factor, and < 0.05 is considered statistically significant.

### Relationship of CARM1 overexpression with the clinicopathologic characteristics of breast invasive carcinoma

When we correlated CARM1 expression with clinicopathologic parameters, we found expression of CARM1 in the nucleus was strongly correlated with the patients’ age at diagnosis (*p* = 0.002), tumor number (*p* <0.001) and tumor grade (*p* = 0.035), whereas it was not associated with other clinical characteristics (Table 1). Spearman correlation analysis was further preformed to confirm the correlation between nuclear CARM1 overexpression and patients’ age, tumor number and grade status, which were −0.197(*p* = 0.002), 0.231(*p* < 0.001) and 0.134(*p* = 0.035) respectively. Cytoplasmic CARM1 expression was only correlated with the patients’ age at diagnosis (*p* = 0.041) and tumor grade (*p* = 0.019, Table 2). Spearman correlation of cytoplasmic CARM1 expression levels to them were −0.130 (*p* = 0.041) and 0.149 (*p* = 0.019), respectively. These results indicated CARM1 overexpression was correlated with poor clinicopathologic characteristics.

### Correlation of hormone receptor, HER2, Ki-67 and p53 with CARM1 overexpression

Staining for CARM1 in the cytoplasm and the nucleus were analyzed separately. We found that in the entire cohort of tumors, CARM1 expression in the nucleus was correlated with ER (*p* = 0.016), PR (*p* = 0.007), HER-2 (*p* = 0.037), Ki-67 (*p* = 0.014) and p53 (*p* = 0.006), which was further confirmed by Spearman correlation analysis. Nuclear expression of CARM1 was negatively correlation with ER (r = −0.153, *p* = 0.016) and PR levels (r = −0.173, *p* = 0.006), and positively correlation with HER-2 (r = 0.132, *p* = 0.038), Ki-67 (r = 0.157, *p* = 0.014) and p53 (r = 0.174, *p* = 0.006). While there was significant difference between cytoplasmic CARM1 expression and expression levels of ER (r = −0.175, *p* = 0.006), PR (r = −0.129, *p* = 0.043), and Ki-67 (r = −0.150, *p* = 0.018). The results suggested overexpression of CARM1 was correlated with aggressive action and poor prognosis.

### Relationship of CARM1 expression with different subtypes of invasive breast carcinoma

To determine the role of CARM1 expression in different subtypes of invasive breast carcinoma, we correlated the nuclear expression of CARM1 with tumor molecular subtypes (Table 3). We found that the nuclear expression of CARM1 was significantly different among four molecular subtypes (*p* = 0.019), while CARM1 expression in the cytoplasm was not (*p* = 0.066). We showed that in the HER-2 subtype, the nuclear expression of CARM1 was the highest (69.6%), followed by the luminal B (59.6%) and basal type (57.1%). The luminal A type showed the lowest percentage of cells with CARM1 nuclear expression (41.3%). Compared with luminal A, luminal B, HER-2, and basal type are correlated with a generally more aggressive tumor phenotype and poorer prognosis [25]. These results suggest that CARM1 overexpression may also be correlated with poor prognosis.

**Table 3:** **Correlation between nuclear**, **cytoplasmic CARM1 expression and molecular subtypes of breast cancer patients**

**Molecular subtypes**	**n**	**Nuclear CARM1 expression**		***P*﹡**	**Cytoplasmic CARM1 expression**		***P*﹡**
		**Negative or low expression (IHC score < 80)**	**High or over expression (IHC score ≥ 80)**		**Negative or low expression (IHC score < 80)**	**High or over expression (IHC score ≥ 80)**	
Lumina A	121	71 (58.7)	50 (41.3)	0.019	60 (49.6)	61 (50.4)	0.066
Luminal B	47	19 (40.4)	28 (59.6)		19 (40.4)	28 (59.6)	
HER2 type	23	7 (30.4)	16 (69.6)		5 (21.7)	18 (78.3)	
triple negative	56	24 (42.9)	32 (57.1)		21 (37.5)	35 (62.5)	

﹡ *P* value is the result of χ^2^ test in the same factor, and < 0.05 is considered statistically significant.

## Discussion

Breast cancer is a heterogeneous disease encompassing multiple subgroups with differing molecular signatures, prognoses, and responses to therapies [27]. Other reasons for heterogeneity may include differences in the studied population (e.g., ethnicity, menopausal status), or it might be due to interaction with other risk factors (e.g., BRCA variants) [28]. El FH found that molecular classification and biological profile may be different according to geographical distribution [29]. Zhang Q’s study showed the ectopic expression of BRCA1 was associated with the genesis, progression, and prognosis in young breast cancer patients [30]. Finding of the sources of heterogeneity would contribute to patient’s stratification and personalized treatment. Our study demonstrated that nuclear CARM1 expression was associated with a younger age at diagnosis; a higher tumor grade; a higher rate of HER2, p53, and ki-67 expression; and a lower rate of ER and PR expression in breast cancer patients of Chinese women. We also found that nuclear CARM1 expression was significant different among four molecular subtypes.

In this study, we demonstrated that CARM1 expression, both in the cytoplasm and the nucleus, were more remarkable in younger patients than in patients older than 40 years of age. Young age at the time of diagnosis of breast cancer is an independent factor of poor prognosis for reasons that are not fully understood [31,32]. Some studies have demonstrated breast cancer at a younger age might be associated with higher grade, ER-negativity, a more advanced stage of the disease, ectopic expression of BRCA1 [30], and higher levels of HER2. Previously, it has been shown that embryonic stem cells overexpressing CARM1 were more resistant to differentiation [33]. In our study, the CARM1 overexpression in younger patients was more common compared with older patients, and might contribute to the clinical characteristics of younger patients, such as lower differentiation. However, we believe it may not be the only factor.

Regarding the subcellular distribution of CARM1, it is predominantly localized in the nucleus as a transcriptional coactivator [34]. That is to say, most of the known functions of CARM1 are related to its nuclear localization. A few studies also revealed that CARM1 accumulates in the cytoplasm during mitosis [14,34]. CARM1 S217E mutant protein and a small percentage of wild-type CARM1 are also localized in the cytosol [34]. This suggested CARM1 may play an unknown function in the cytoplasm [34]. Because nucleus is the principal subcellular localization of CARM1 activation, we will focus on nuclear CARM1 expression hereinafter.

Our results indicated that CARM1 expression positively correlated with HER2 expression and grade, and negatively correlated with hormone receptors in separate analyses with universal molecular makers. The molecular subtypes were also classified according to a panel of ER, PR, and HER2 biomarkers combined with grade in our study [31]. We will discuss the correlation between these factors and CARM1 expression in different molecular subtypes.

The luminal A subtype, known as the hormone subtype, showed the lowest rate of CARM1 expression compared with that in the other subtypes. Previous reports have shown that CARM1 plays an essential role in estrogen-mediated transcriptional activation [35,36], and is necessary for the estradiol (E2)-induced proliferation of breast cancer cells [15]. The co-regulator requirement of CARM1 can be highly tissue- and context-dependent [8,13,37]. Furthermore, CARM1 transcriptional coactivating functions are not restricted to nuclear receptors [16,33]. Consistent with this concept, our result also shows only a small part of luminal A subtype cancer tissue overexpress nuclear CARM1.

Our study demonstrated that CARM1 overexpression in breast cancer was associated with the overexpression of HER2. Both HER-2 subtype (69.6%) and luminal B subtype (59.6%) showed higher rate of CARM1 expression compared with that in luminal A tumors. Within luminal B subtype, HER2 signaling is dominant, as demonstrated by the poor response of such tumors to endocrine therapy alone. HER2 overexpression confers intrinsic or primary resistance to hormone-based therapy despite the presence of hormone receptors [38,39]. In this study, we showed that there was a strong positive correlation between CARM1 and HER2 expression. This suggests that CARM1 may be useful as a predictor of clinical outcomes in patients with HER2-positive tumors.

The mechanism of CARM1 and HER2 interaction, or through which pathway they crosstalk, has not yet been elucidated. One potential mechanism may be the transcriptional coactivation mediated by CARM1 and p160. CARM1 was initially described as a transcriptional coactivator of the p160 nuclear receptor family [8]. All members of the p160 family are natural substrates of CARM1, which can bind and recruit CARM1 to synergistically exert transcriptional co-activating functions of target genes [40]. Multiple studies have demonstrated that AIB1 (a member of the p160 family) mRNA and protein expression in breast cancer is associated with the expression of HER2. AIB1 was shown to play a role in the regulation of the HER2 pathway [41]. Although AIB1 expression was not examined in our study, data from El Messaoudi S et al. showed that AIB1 and CARM1 mRNA levels were both elevated in breast cancer, notably in grade 3 [16]. Our finding that CARM1 overexpression in breast cancer correlated with high HER2 expression supports the hypothesis that CARM1 might play an important role in the regulation of the HER2 pathway, probably through transcriptional coactivation with the p160 family.

TN breast cancer is associated with poor prognosis because it lacks the benefit of specific therapy. The TN subtype group encompasses a number of distinct entities with defined gene expression profiles and outcomes [39]. Our results revealed that over half of the TN tumors overexpressed nuclear CARM1. These results suggest that monitoring the level of CARM1 expression might be valuable to distinguish different entities of TN tumors. Future research should explore this hypothesis as well as its clinical applicability

In summary, based on previous work and the results presented here, CARM1 may promote tumor cell growth by activating nuclear receptors and multiple growth factor signaling cascades in breast cancer. However, the predominance of which pathway is regulated by CARM1 depends on the tumor phenotype. CARM1 requires its enzymatic activity for all of its known nuclear functions. Thus, specific and potent small molecule inhibitors of CARM1 will incapacitate all of its nuclear functions [42]. Additionally, chemotherapeutic drugs targeted at CARM1 will likely interfere with several pathways concomitantly. Therefore, targeted therapy of CARM1 is promising and warrants further exploration.

One limitation of the study is that we didn’t know the relationship of CARM1 overexpression and the prognosis of breast cancer. It still remains unclear about the accurate regulative pathways mentioned above in cancer cells. All these need to be explored by further study.

## Conclusions

In this study, we demonstrated that CARM1 expression was increased in invasive breast cancer cells compared with adjacent benign epithelium. Nuclear CARM1 expression was associated with a younger age at diagnosis; multi-center origin or multiple tumors; a higher tumor grade; a higher rate of HER2, p53, and Ki-67 expression; and a lower rate of ER and PR expression. All of these predictors are clinicpathologic parameters that correlate with poor prognosis. This suggests that CARM1 might have the potential to improve the stratification and personal management of patients suffering from breast cancer. We also found that the rate of CARM1 expression was significantly different among different molecular subtypes of breast cancer, which may hint at a different mechanism and prognosis value. This should be further investigated.

## Abbreviations

CARM1: Coactivator-associated arginine methyltransferase 1; ER: Estrogen receptor; PR: Progesterone receptor; HER: Epidermal growth factor receptor; PRMTs: Methylation of histones by protein arginine methyltransferases; SRC: Steroid receptor coactivators-1; AIB1: Amplified in breast cancer 1; NRs: Nuclear receptors; IHC score: Immunohistochemical score; TN: Triple negative.

## Competing interests

The authors declare no conflict of interest.

## Authors’ contributions

HC collected clinical data, evaluated the immunohistochemical stainings, performed the statistical analyses and drafted the manuscript. YQ assisted with the design of the study, evaluation of the immunohistochemical stainings and pathological diagnosis. HF carried out the immunoassays and fluorescent in situ hybridization analysis. PS assisted with the immunoassays and the collection of clinical data. XZ and HZ were involved in pathological diagnosis and evaluated the immunohistochemical stainings. GZ conceived the study, was involved in the design, and edited the manuscript for intellectual content. All authors read and approved the final manuscript.
